# Biosynthetic pathway for leukotrienes is stimulated by lipopolysaccharide and cytokines in pig endometrial stromal cells

**DOI:** 10.1038/s41598-025-86787-1

**Published:** 2025-01-22

**Authors:** Barbara Jana, Aneta Andronowska, Jarosław Całka, Aleksandra Mówińska

**Affiliations:** 1 Division of Reproductive Biology, Institute of Animal Reproduction and Food Research, Polish Academy of Sciences, Tuwima 10, Olsztyn, 10-748 Poland; 2 Department of Clinical Physiology, Faculty of Veterinary Medicine, University of Warmia and Mazury, Oczapowskiego 13, Olsztyn, 10-719 Poland

**Keywords:** Endometrium, Stromal cells, Lipopolysaccharide, Cytokines, Leukotriene synthesis/Release, Pig, Reproductive disorders, Mechanisms of disease, Cytokines

## Abstract

An inflammatory response is related to different inflammatory mediators generated by immune and endometrial cells. The links between lipopolysaccharide (LPS), cytokines, and leukotrienes (LTs) in endometrial stromal cells remain unclear. This study aimed to examine the influence of LPS, tumor necrosis factor (TNF)-α, interleukin (IL)-1β, IL-4 and IL-10 on 5-lipooxygenase (5-LO), LTA4 hydrolase (LTAH) and LTC4 synthase (LTCS) mRNA and protein abundances, and LTB4 and cysteinyl (cys)-LTs release including LTC4, by the cultured pig endometrial stromal cells, as well as on cell viability. 24-hour exposure to LPS, TNF-α, IL-4 and IL-10 up-regulated 5-LO mRNA and protein abundances. LPS increased LTAH mRNA abundance, while TNF-α, IL-1β and IL-10 augmented LTAH mRNA and protein abundances. TNF-α and IL-4 increased LTCS mRNA and protein abundances. In addition, LTCS mRNA abundance was enhanced by LPS and IL-4, while LTCS protein abundance was increased by IL-1β. Cells responded to LPS, TNF-α, IL-1β and IL-10 with increased LTB4 release. TNF-α, IL-1β and IL-4 stimulated LTC4 release. Cys-LTs release was up-regulated by LPS, TNF-α, IL-1β and IL-4. All studied cytokines augmented cell viability. In summary, LPS, TNF-α, IL-1β, IL-4 and IL-10 are potential LTs immunomodulatory agents in endometrial stromal cells. These functional interactions could be one of the mechanisms responsible for local orchestrating events in inflamed and healthy endometrium.

## Introduction

Endometritis is considered a significant global problem in animals and is associated with reproductive disorders and huge economic losses^1,2^. In women, endometritis is a common disease of the reproductive tract which can lead to a number of adverse health effects, including infertility^3^. The occurrence of uterine inflammation depends on several factors, such as the host’s innate immune response, the species and the number of microorganisms colonizing the endometrium and uterine contractility. Inflammation of the uterus is often associated with many different bacterial infections, including those evoked by Gram-negative bacteria^4,5^. Lipopolysaccharide (LPS), a component of the outer membrane of Gram-negative bacteria, is widely used as an efficient immune stimulus both in *vivo* and in vitro conditions. LPS, acting through the Toll-like receptor 4 located on endometrial cells, can activate the formation of many downstream inflammatory mediators and aggregate the occurrence and development of the inflammatory process^6–9^. In the inflamed uterus, considerable amounts of tumor necrosis factor (TNF)-α, interleukin (IL)-1β, IL-6 and IL-8 having pro-inflammatory effects, as well as IL-10 with anti-inflammatory properties are generated^10–12^.

Literature data show the significance of TNF-α, IL-1β, IL-4 and IL-10 and their receptors for female reproductive processes. For example, it is known that TNF-α receptors are present in endometrial cells, including stromal cells^13^ and that this cytokine plays a significant role in embryo implantation^14^. The importance of IL-1β and its receptors was revealed in the parturition^15^. Moreover, IL-1β and IL-4 (also IL-2) show differential expression during pregnancy and at term, suggesting their role in creating the inflammatory state of these periods^16^. In turn, IL-10 induces a pro-fibrotic phenotype of endometriotic stromal cells^17^.

Leukotrienes (LTs) generation from arachidonic acid is started by 5-lipoxygenase (5-LO), which requires 5-LO activating protein. Formed LTA4 is then hydrolyzed by LTA4 hydrolase (LTAH) to produce LTB4 or conjugated with reduced glutathione by LTC4 synthase (LTCS) to yield LTC4. This LT is the first stage to generate cysteinyl-LTs (cys-LTs): LTC4, LTD4, LTE4^18^. With regard to the LT biosynthesis pathway in the uterus with inflammation, it is known that this process in cows and pigs clearly increased the secretion of LTB4 and LTC4^19–21^. In an inflamed pig endometrium, the contents of both LTs were convergent with the rise in the expression of 5-LO, LTAH and LTCS^21^.

The endometrium is composed of epithelial, endothelial, stromal cells and various immune cells interacting with each other in a paracrine manner, which plays an important role in physiological and pathological processes. An inflammatory state in the endometrium originates and is maintained by interactions between factors released by the different types of endometrial cells^8,22^. To date, the links between cytokines having pro- and anti-inflammatory activity and LTs, generated in large quantities by endometrial cells in response to inflammatory factors, as well as in healthy uteruses, are not completely recognized. The authors reported earlier that LPS, TNF-α, IL-1β, IL-4 and IL-10 stimulate the LTAH and LTCS expression and LTB4 and LTC4 secretion from the pig healthy and inflamed endometrium^23^. In addition, LPS, TNF-α, IL-1β and IL-10 influence 5-LO, LTAH and LTCS expression and LTB4 and/or LTC4 secretion by pig endometrial epithelial^24^ and endothelial^25^ cells. Thus, it is hypothesized that LPS, TNF-α, IL-1β, IL-4 and IL-10 change the LTB4 and LTC4 production and release by the endometrial stromal cells. Investigating these relationships will help to better understand the immunological mechanisms that take place during uterine inflammatory and physiological conditions. The obtained results may contribute to improving the prevention and treatment rates of uterine inflammation in animals and women. The current article analyzed the influence of LPS, TNF-α, IL-1β, IL-4 and IL-10 on 5-LO, LTAH and LTCS mRNA and protein abundances in the cultured pig endometrial stromal cells, LTB4 and cys-LTs release including LTC4, by these cells and on cell viability.

## Results

### Influence of inflammatory factors on 5-LO mRNA and protein abundances in cultured endometrial stromal cells

**Fig. 1:**
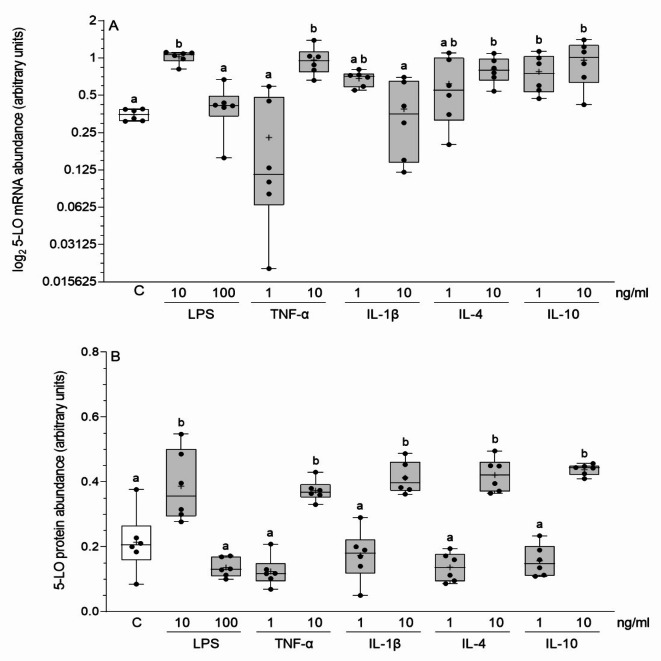
Influence of lipopolysaccharide (LPS), tumor necrosis factor-α (TNF-α), interleukin (IL)-1β, IL-4 and IL-10 on 5-lipoxygenase (5-LO) mRNA (**A**) and protein (**B**) abundances in the cultured endometrial stromal cells of pigs. Box plots show individual results from Real-Time PCR and Western blotting in relation to glyceraldehyde-3-phosphate dehydrogenase (GAPDH). The line depicts the median value and the cross depicts mean value within a box. Data were obtained from six pigs. Treatments were conducted in triplicate for each pig. Representative protein bands for each treatment are presented on blots (Supplementary Fig. 1A). Different letters (a, b) above the boxes indicate significant differences (*P* < 0.05 − 0.001). The same letter above boxes indicates no significant difference. C – control (vehicle-treated cells).

The 5-LO mRNA abundances in the stromal cells were higher after using 10 ng/ml of LPS (*P* < 0.001), TNF-α (*P* < 0.01) and IL-4 (*P* < 0.05), and 1 and 10 ng/ml of IL-10 (*P* < 0.05) when compared to the control (vehicle-treated cells) (Fig. 1A). The 5-LO protein abundances were increased after treatment with the smaller dose of LPS (*P* < 0.001), larger doses of TNF-α (*P* < 0.01), IL-1β, IL-4 and IL-10 (*P* < 0.001) (Fig. 1B). A statistical comparison of the influence of particular inflammatory mediators on the 5-LO mRNA and protein abundances is depicted in Fig. 1A and B.

### Influence of inflammatory factors on LTAH mRNA and protein abundances and LTB4 release by cultured endometrial stromal cells

**Fig. 2:**
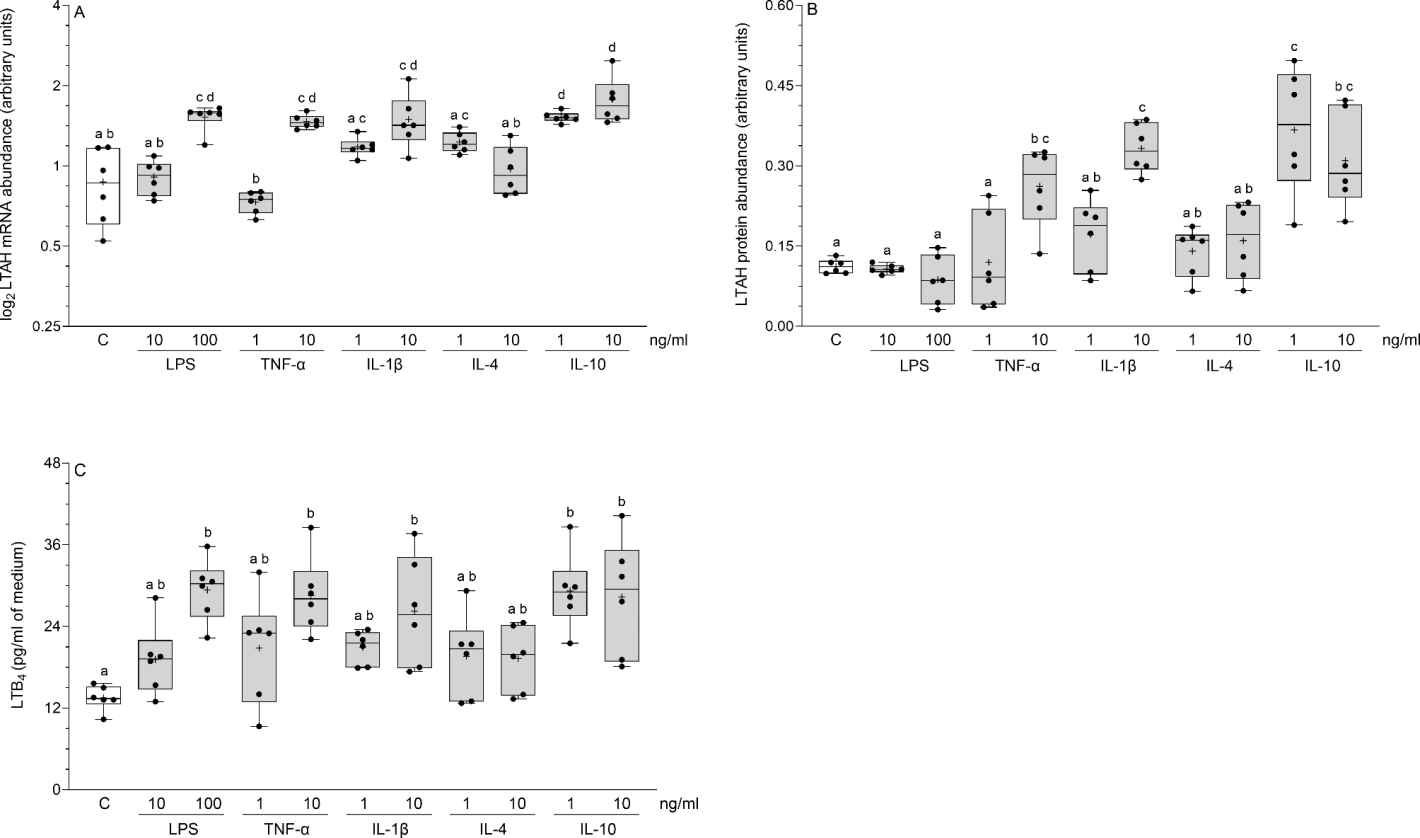
Influence of lipopolysaccharide (LPS), tumor necrosis factor-α (TNF-α), interleukin (IL)-1β, IL-4 and IL-10 on leukotriene A4 hydrolase (LTAH) mRNA (**A**) and protein (**B**) abundances and on leukotriene (LT)B4 (**C**) secretion by the cultured endometrial stromal cells of pigs. Box plots show individual results from Real-Time PCR and Western blotting in relation to glyceraldehyde-3-phosphate dehydrogenase (GAPDH) and from ELISA. The line depicts the median value and the cross depicts mean value within a box. Data were obtained from six pigs. Treatments were conducted in triplicate for each pig. Representative protein bands for each treatment are presented on blots (Supplementary Fig. 1B). Different letters (a, b, c, d) above boxes indicate significant differences (*P* < 0.05 − 0.001). The same letter above boxes indicates no significant difference. C – control (vehicle-treated cells).

In the stromal cells, the LTAH mRNA abundances were increased (*P* < 0.001) in response to the larger doses of LPS, TNF-α and IL-1β, and both doses of IL-10 (Fig. 2A). The larger doses of TNF-α (*P* < 0.05), IL-1β (*P* < 0.001), and both doses of IL-10 (*P* < 0.001) increased LTAH protein abundances (Fig. 2B). The concentrations of LTB4 in the medium of cultured stromal cells were increased after exposure to the larger doses of LPS, TNF-α (*P* < 0.01) and IL-1β (*P* < 0.05), and both doses of IL-10 (*P* < 0.01) (Fig. 2C). A statistical comparison of the influence of particular inflammatory mediators on the LTAH mRNA and protein abundances and LTB4 release is depicted in Fig. 2A-C.

### Influence of inflammatory factors on LTCS mRNA and protein abundances and LTC4 and cys-LTs release by cultured endometrial stromal cells

**Fig. 3:**
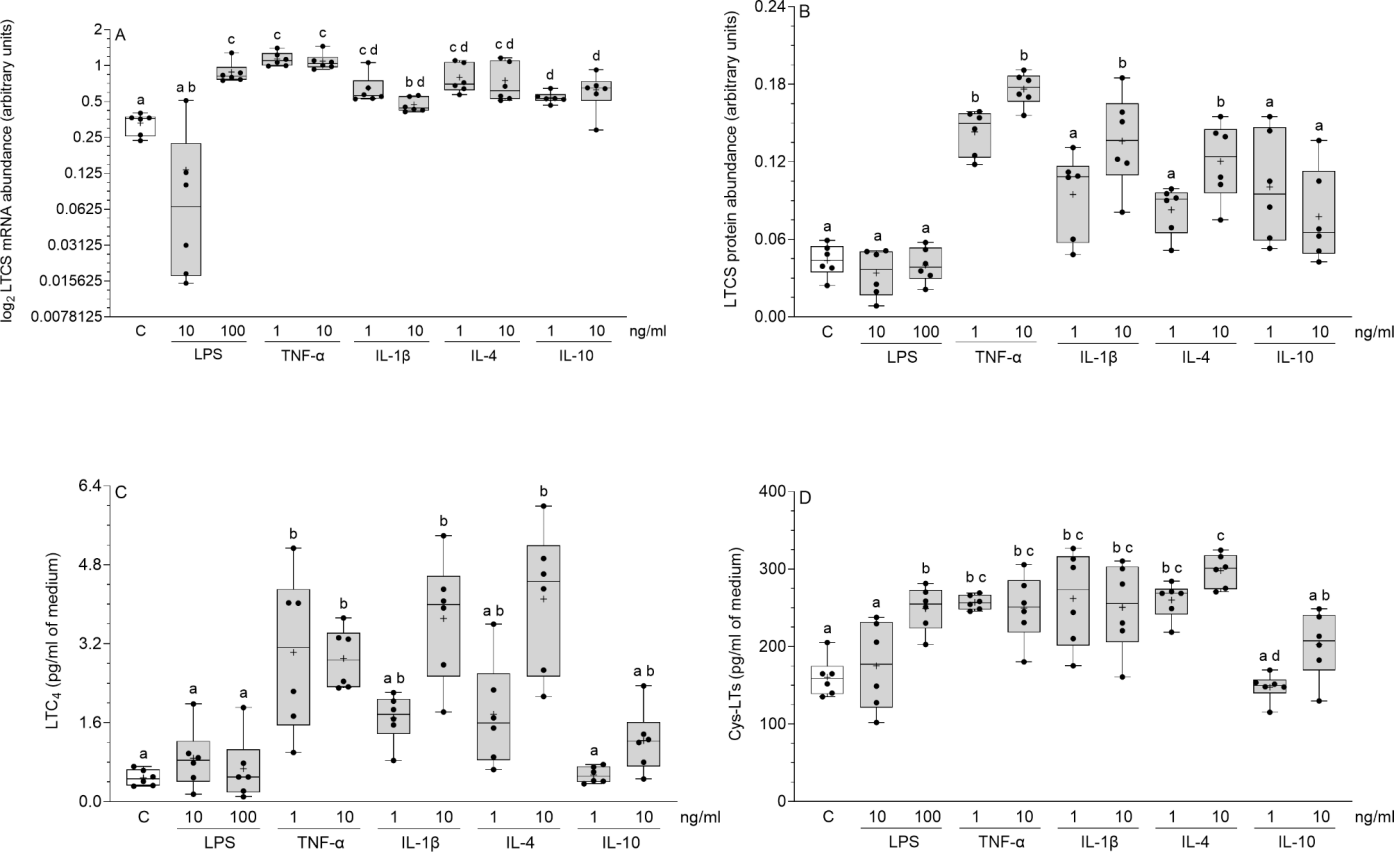
Influence of lipopolysaccharide (LPS), tumor necrosis factor-α (TNF-α), interleukin (IL)-1β, IL-4 and IL-10 on leukotriene C4 synthase (LTCS) mRNA (**A**) and protein (**B**) abundances and on leukotriene (LT)C4 (**C**) and cysteinyl-LTs (cys-LTs; **D**) secretion by the cultured endometrial stromal cells of pigs. Box plots show individual results from Real-Time PCR and Western blotting in relation to glyceraldehyde-3-phosphate dehydrogenase (GAPDH) and from ELISA. The line depicts the median value and the cross depicts mean value within a box. Data were obtained from six pigs. Treatments were conducted in triplicate for each pig. Representative protein bands for each treatment are presented on blots (Supplementary Fig. 1C). Different letters (a, b, c, d) above boxes indicate significant differences (*P* < 0.05 − 0.001). The same letter above boxes indicates no significant difference. C – control (vehicle-treated cells).

After the application of the larger dose of LPS (*P* < 0.001), both doses of TNF-α (*P* < 0.001) and IL-4 (smaller dose: *P* < 0.01, larger dose: *P* < 0.05), the LTCS mRNA abundances in the stromal cells were augmented (Fig. 3A). The smaller and larger doses of TNF-α and the larger doses of IL-1β and IL-4 enhanced (*P* < 0.001) the LTCS protein abundances (Fig. 3B). Both doses of TNF-α (*P* < 0.01) and the larger doses of IL-1β and IL-4 (*P* < 0.001) all stimulated the LTC4 release from the stromal cells (Fig. 3C). The concentrations of cys-LTs in the medium were higher after treatment with the larger dose of LPS (*P* < 0.05), both doses of TNF-α (smaller dose: *P* < 0.01, larger dose: *P* < 0.05), IL-1β (*P* < 0.01) and IL-4 (smaller dose: *P* < 0.01, larger dose: *P* < 0.001) (Fig. 3D). A statistical comparison of the influence of particular inflammatory mediators on the LTCS mRNA and protein abundances and LTC4 and cys-LTs release is depicted in Fig. 3A-D.

### Influence of inflammatory mediators on the viability of cultured endometrial stromal cells

**Fig. 4:**
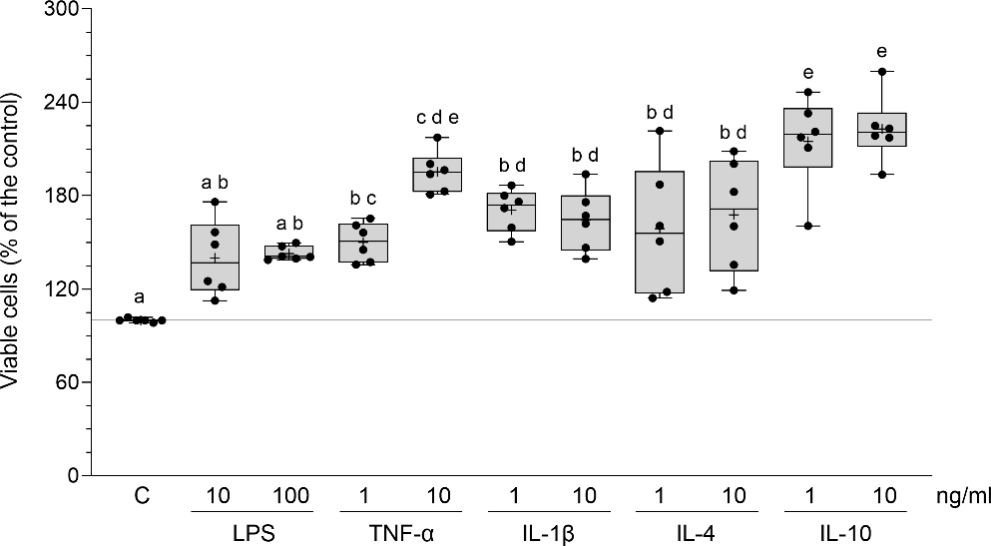
Influence of lipopolysaccharide (LPS), tumor necrosis factor-α (TNF-α), interleukin (IL)-1β, IL-4 and IL-10 on the cultured endometrial stromal cell viability of pigs. Box plots show individual results from MTT assay. The line depicts the median value and the cross depicts mean value within a box. Data were obtained from 6 pigs. Treatments were conducted in triplicate for each pig. The viability of control (vehicle-treated cells, C) determined by absorbance at a wavelength of 570 nm was 0.34 ± 0.007. This value was taken as 100% cell viability, and the effects of mediators were normalized to that. Different letters (a, b, c, d, e) above boxes indicate significant differences (*P* < 0.05 − 0.001). The same letter above boxes indicates no significant difference.

The viability and proliferation potential of endometrial stromal cells were increased after exposure to both doses of TNF-α (smaller dose: *P* < 0.05, larger dose: *P* < 0.001), IL-1β (*P* < 0.001), IL-4 (smaller dose: *P* < 0.01, larger dose: *P* < 0.001) and IL-10 (*P* < 0.001) (Fig. 4). A statistical comparison of the influence of particular inflammatory mediators on the cell viability is depicted in Fig. 4.

The results concerning the synthesis and secretion of LTs from the cultured endometrial stromal and the viability of these cells after exposure to the applied inflammatory mediators are summarized in Table 1.

**Table 1:** Summary of the results obtained on the effects of lipopolysaccharide (LPS), tumor necrosis factor-α (TNF-α), interleukin (IL)-1β, IL-4 and IL-10 on 5-lipoxygenase (5-LO), leukotriene A4 hydrolase (LTAH) and leukotriene C4 synthase (LTCS) mRNA and protein abundances, and leukotriene (LT)B4, LTC4 and cys-LTs release by the cultured porcine endometrial stromal cells, as well as on these cell viability. Concentrations of the applied mediators are given in ng/ml of medium.

Determined parameters		LPS		TNF-α		IL-1β		IL-4		IL-10	
		10	100	1	10	1	10	1	10	1	10
5-LO abundance:	mRNA	↑	-	-	↑	-	-	-	↑	↑	↑
	protein	↑	-	-	↑	-	↑	-	↑	-	↑
LTAH abundance:	mRNA	-	↑	-	↑	-	↑	-	-	↑	↑
	protein	-	-	-	↑	-	↑	-	-	↑	↑
LTB4 secretion		-	↑	-	↑	-	↑	-	-	↑	↑
LTCS abundance	mRNA	-	↑	↑	↑	-	-	↑	↑	-	-
	protein	-	-	↑	↑	-	↑	-	↑	-	-
LTC4 secretion		-	-	↑	↑	-	↑	-	↑	-	-
Cys-LTs secretion		-	↑	↑	↑	↑	↑	↑	↑	-	-
Cell viability		-	-	↑	↑	↑	↑	↑	↑	↑	↑

↑- indicates a statistically significant increase of values of the studied parameters after exposure to the applied mediators versus the control (vehicle-treated cells);

- - indicates a lack of statistically significant effect.

## Discussion

Endometrial stromal cells, along with epithelial cells, are involved in the immune response because they express pattern recognition receptors for the detection of microbes and produce a classic inflammatory response to bacteria^6^. Moreover, next to the epithelial and endothelial cells of the pig healthy and inflamed endometrium, the immunoexpression for 5-LO, LTAH and LTCS was evident in the stromal cells indicating that these cells may be also the source of LTs^21^. However, the significance of stromal cells in the pathogenesis of endometritis in terms of the regulation of the generation of metabolites derived from the 5-LO pathway by inflammatory mediators is unknown. It is also known that there is a delicate balance between the pro- and anti-inflammatory response. Pro-inflammatory mediators act to initiate and increase the response, while anti-inflammatory cytokines counteract this^26^. Therefore, the present study was undertaken to reveal how pro- (LPS, TNF-α, IL-1β) and anti- (IL-4, IL-10) -inflammatory mediators affect LTB4 and LTC4 formation and cys-LTs release, including LTC4, from the cultured porcine endometrial stromal cells, as well as the viability of these cells.

Previous findings show that LPS up-regulated 5-LO, LTAH and LTCS expression, as well as LTB4 and LTC4 secretion by the human bronchial epithelial cells^27^. In turn, TNF-α with interferon-γ increased LTCS and LTAH mRNA expression and LTC4 release from the bovine luteal endothelial cells, but no significant effect of these cytokines on LTAH protein expression and LTB4 release was noted^28^. In the bovine mammary gland tissues^29^ and epithelial cells^30^, LPS, TNF-α and IL-1α stimulated LTB4 and LTC4 release. Moreover, these cells responded to TNF-α with increased 5-LO mRNA expression. In reference to the uterus, it is known that LPS, TNF-α and IL-1β are able to enhance LTAH and LTCS expression and LTB4 and LTC4 secretion by explants from the pig healthy and inflamed endometrium^23^. In addition, 5-LO, LTAH and LTCS mRNA and/or protein expression and LTB4 and LTC4 secretion by the pig endometrial epithelial^24^ and endothelial^25^ cells were increased when challenged with these pro-inflammatory mediators, except for the effect of IL-1β on LTB4 release by the endothelial cells. The authors’ recent findings indicate an increase in mRNA and/or protein abundances of 5-LO, LTAH, and LTCS following the exposure of endometrial stromal cells to LPS, TNF-α, and IL-1β, which generally supports the previous data. In line with earlier studies^23–25^, the current study also found that changes in enzyme abundances were accompanied by a rise in the secretion of LTB4 and LTC4 from the stromal cells, excluding the LPS effect on LTC4 release. The important observation of the current study is that there was an increase in the cys-LTs released by stromal cells exposed to the pro-inflammatory mediators used. A comparison of the values of cys-LTs with LTC4 secreted by the stromal cells in response to LPS, TNF-α and IL-1β showed a significant advantage of cys-LTs. This indicates a possible stimulatory effect of pro-inflammatory factors not only on the release of LTC4 but also on other cys-LTs, i.e., LTD4 and LTE4.

In regard to the anti-inflammatory activities of IL-4 and IL-10 in the endometrial stromal cells, literature data show that both cytokines inhibit TNFα-stimulated chemokine generation^31^ and IL-10 reduces TNFα-induced IL-6 production^32^. The results of the present research indicate that the actions of IL-4 and IL-10 on the formation and release of particular LTs from the porcine endometrial stromal cells vary. IL-4 increased the 5-LO and LTCS mRNA and protein abundances and cys-LTs secretion, including LTC4. This is in line with the results regarding LTC4 synthesis and release from the porcine endometrial endothelial cells^25^ and human mast cells^33^. It was also found that IL-4 did not significantly change LTB4 release by the stromal cells, which is consistent with the findings obtained in the endothelial cells^25^. Although IL-4 did not significantly change the LTAH abundance in the current study, this enzyme was reduced in the endothelial cells^25^ and increased in the epithelial cells^24^ by this cytokine. IL-4 did not significantly change LTAH expression or LTB4 release by monocytes^34^. Both IL-4 and IL-13 up-regulated LTB4 production in neutrophils^35^. In turn, the present study found that IL-10 exerted a stimulatory effect on 5-LO and LTAH mRNA and protein abundances and LTB4 release by the pig endometrial stromal cells. In contrast, this cytokine did not significantly change the LTCS abundance or cys-LTs secretion, including LTC4. Previous studies found that the treatment with IL-10 led to increased production and release of LTB4 and LTC4 by porcine endometrial epithelial^24^ and endothelial^25^ cells, as well as explants^23^. However, the current study found that the actions of IL-10 on the 5-LO pathway enzymes and LTB4 release by the stromal cells are opposite to those revealed in dendritic cells^36^.

As reported in the present study, the excitatory effect of pro- (LPS, TNF-α, IL-1β) and anti (IL-4, IL-10) -inflammatory mediators on the LTB4 and/or LTC4 production and/or cys-LTs release, including LTC4 by the endometrial stromal cells suggests that these mediators may indirectly influence endometrial processes modulated by LTs. Regarding the significance of LTs in the uterine secretory activity, research have shown that both LTB4 and LTC4 augment the release of prostaglandin (PG)E2, PGF2α and IL-6 from *E. coli-*exposed bovine uterine explants^22^. Additionally, these LTs increased PGE2 and PGF2 secretion from healthy bovine endometrium^37^. The role of LTC4 and LTD4 in the contractile activity of healthy and inflamed porcine uterus was also reported^38^. Moreover, literature data show the participation of LTs in the inflammatory process. During endometritis, LTB4 enhances chemotaxis and the random migration of neutrophils into the uterine lumen^20^. In turn, LTC4, besides contributing to the development of inflammation, also participates in the maintenance and regeneration of damaged tissues^39^. Based on the present study, it is also supposed that the anti-inflammatory action of IL-4 in the porcine endothelial stromal cells may be due to its negligible impact on LTB4 production and release. However, the anti-inflammatory properties of IL-10 may be a consequence of its limited effect on LTC4 synthesis and cys-LT release (including LTC4). The current study suggests that the regulation of LTB4 and/or LTC4 production, as well as the release cys-LTs including LTC4, by endometrial stromal cells in response to pro- and anti-inflammatory factors, may play a significant role in uterine function. This regulation could also be important for the origin, development, and maintenance of inflammation.

Moreover, the pig endometrial stromal cells responded to TNF-α, IL-1β, IL-4 and IL-10 with increased viability and proliferation. TNF-α and IL-4 actions are consistent with those noted in the porcine endometrial epithelial^24^ and endothelial^25^ cells. TNF-α also up-regulated the viability of human endometriotic stromal cells^40^ and the equine endometrial^41^ and bovine mammary gland^30^ epithelial cells, while IL-4 increased the proliferation of human mast cells^33^. It was reported that IL-1β increased the pig endometrial endothelial cell viability and proliferation^25^ and that IL-1α augmented the proliferation of the equine endometrial cells^42^. Similar to the present study, IL-10 induced the viability of human decidual stromal cells^43^ and pro-fibrotic phenotype, including cell proliferation in the human endometriotic stromal cells^17^. In contrast, IL-10 did not significantly affect the viability and proliferation of the porcine endometrial epithelial^24^ and endothelial^25^ cells. In addition, the current study found that LPS (1 or 10 ng/ml of medium) did not significantly affect stromal cell viability, similarly as in the endometrial epithelial^24^ and endothelial^25^ cells. These findings disagree with reports that demonstrated inhibition in the cell viability and proliferation of goat^44^ and human decidual^45^ stromal cells exposed to LPS at doses of 20–10 µg/ml of medium, respectively. Thus, the effect of LPS on the endometrial stromal cell viability and proliferation may be dose-dependent. It is possible that the increased viability of the endometrial stromal cells by TNF-α, IL-1β, IL-4 and IL-10 found in the present study may be important for endometrium function. The stromal cells are crucial for the establishment and maintenance of pregnancy^46,47^, and the release of paracrine factors regulates the development, differentiation, and homeostasis of the epithelium^48^. It is possible that TNF-α, IL-1β, IL-4 and IL-10 significantly generated during endometritis, by increasing the viability of stromal cells, may affect the regeneration of endometrial cells damaged by inflammation.

Literature data show that the 5-LO, LTAH and LTCS expression in the porcine endometrial and/or myometrial layers^21^, as well as the horse^49^ and bovine^37^ endometrium, changes at different stages of the estrous cycle. During the ovarian cycle, endometrial estrogen and progesterone receptor expression also vary^50,51^, including immunostaining patterns for these receptors in the stromal cells^52^. Thus, it is possible that, in the present study, the generation and release of LTB4 and/or cys-LT after exposure to LPS, TNF-α, IL-1β, IL-4 and IL-10, as well as the effects of cytokines on endometrial stromal cell viability and proliferation, may be controlled by the ovarian steroids.

## Conclusions

This study is the first to show that LPS, TNF-α and IL-1β increase the production and secretion of LTB4 and cyst-LTs by the pig endometrial stromal cells. In turn, IL-4 stimulates LTC4 generation and cyst-LTs release, including LTC4, while IL-10 enhances LTB4 production and release. TNF-α, IL-1β, IL-4 and IL-10 augmented the cell viability. These facts suggest the possibility that LPS and cytokines controlling LT formation and release by endometrial stromal cells indirectly affect the functions of these cells. Activation of the 5-LO pathway in the stromal cells by pro- and anti-inflammatory mediators could be one of the mechanisms responsible for endometrial local orchestrating events under inflammatory and physiological conditions. Moreover, the links determined in the current study between LPS, cytokines and LTs in the cultured endometrial stromal cells of pigs indicate that this cellular model may be used to better understand the immune mechanisms occurring under inflammatory and physiological conditions in the uterus.

## Materials and methods

### Animals and uterine tissue collection

Uteri from gilts (*n* = 6) were harvested from a local abattoir. Entire uteri were collected within 5 min after the gilt slaughter. To determine the phase of the estrous cycle, a macroscopic assessment of the ovaries was performed^53^. Uteri on days 6–8 of the estrous cycle were collected for the experiment. Cells isolated from uteri taken on these days of the estrous cycle easily become confluent in culture conditions. The uteri were kept on ice and immediately transported to the laboratory.

### Isolation of endometrial stromal cells

**Fig. 5:**
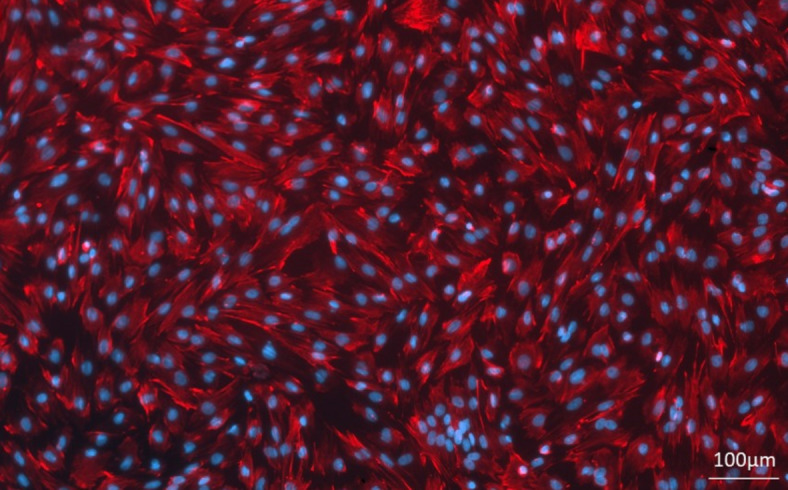
The purity of endometrial stromal cell culture confirmed by immunofluorescent staining with anti-vimentin antibodies (red); cell nuclei stained with DAPI (blue). Scale bar: 100 μm.

Pieces of both porcine uterine horns (5 cm in length) were washed twice with sterile phosphate-buffered saline (PBS; NaCl, KCl, Na2HPO4 and KH2PO4, pH 7.4) supplemented with antibiotics (100 IU/ml penicillin and 100 g/ml streptomycin; cat. no. 15140–122; Life Technologies, The Netherlands). Endometrial tissue was separated from the myometrium, minced into small pieces and digested for 80 min in 0.06% collagenase type I (cat. C2674, Sigma Aldrich, Germany) in phenol red-free Medium 199 (cat. no. M3769, Sigma) with continuous stirring. Undigested tissue was removed by filtration through a 200 μm mesh strainer. The collected supernatant was mixed with Medium 199 supplemented with 5% (w/v) newborn calf serum (NCS; cat. no. N4637, Sigma) and antibiotics and centrifuged (10 min, 15 °C, 1100 rpm). Erythrocytes were removed by 10 s pipetting the pellet with ice-cold-sterile dH_2_O. The cell washing and centrifugation was repeated three times. Cells were counted and seeded onto 24-well plates (5 × 10^4^ cells per well) and cultured in Medium 199 with 10% NCS and antibiotics until they reached 70% confluence at 37 °C in a humified atmosphere of 95% air and 5% CO_2_. The purity of stromal cells was confirmed by immunofluorescent staining with anti-vimentin antibodies (cat. no. sc-6260, Santa Cruz Biotechnology). The homogeneity of the cell populations ranged from 90 to 100% (Fig. 5).

### Incubation of endometrial stromal cells with LPS and cytokines

The stromal cells were rinsed with fresh culture medium and treated with phenol red-free culture medium containing 2% BSA, 10% NCS and antibiotics without stimulatory factors (control, non-treated cells) or with the application of the following stimulatory factors − 10 or 100 ng/ml of LPS (cat. no. L2880) and 1 or 10 ng/ml of TNF-α (cat. no. T6674), IL-1β (cat. no. SRP3083), IL-4 (cat. no. SRP4137) and/or IL-10 (cat. no. SRP3071), for 24 h. The supplier of all factors was Sigma. To control the reactivity of endometrial stromal cells, the release of PGF2α after exposure to nitric oxide (NO) donor NONOate (at a dose of 10^− 4^ M; cat. no. 82150, Cayman Chemical Co.) was determined. The doses of LPS, cytokines and NONOate, as well as the time of cell culture, were determined in the preliminary studies (Supplementary Figs. 2 and 3). Each treatment was performed in triplicate using cells originating from particular animals (*n* = 6). After the incubation period, the conditioned culture medium was collected into tubes with 5 µl EDTA (cat. no. 879810429, POCH, Gliwice, Poland), 1% acetylsalicylic acid solution (cat. no. A2093, Sigma) and frozen at -20^o^C for EIA method. The cells were then lysed with Fenozol buffer (cat. no. K00203-1, A&A Biotechnology, Gdansk, Poland) and stored at -80^o^C for real-time reverse transcriptase-polymerase chain reaction (real-time RT-PCR). The stromal cells were washed with PBS and lysed with 240 µl of ice-cold RIPA buffer (50 mmol/l Tris–HCl, pH 7.4 /cat. no. T1503, Sigma/; 150 mmol/l NaCl /cat. no. 794121116, POCH, Gliwice, Poland/; 1% Triton X-100 (v/v) /cat. no. T8787, Sigma/; 0.5% sodium deoxycholate (w/v) /cat. no. D6750, Sigma/; 0.1% sodium dodecyl sulphate (w/v) /SDS, cat. no. L3771, Sigma/; 1 mmol/l EDTA) together with protease inhibitor cocktail (cat. no. P8340, Sigma) and centrifuged for 5 min at 800 x *g.* The supernatant was placed at -80^o^C for Western blot analysis. The stromal cells were also used to determine their viability using the MMT method.

### RNA extraction and real-time RT-PCR

Total RNA was isolated with the Total RNA Prep Plus kit (A&A Biotechnology) and treated with DNase I (Life Technology) according to the supplier’s instructions to detect any possible contamination with DNA. Before use, RNA purity and quality were estimated spectrophotometrically using absorbances at 260 and 280 nm, and by agarose gel electrophoresis. Each sample (1 µg) of total RNA was reverse-transcribed into cDNA using QuantiTect Reverse Transcription (cat. no. 205311, QIAGEN, Hilden, Germany), as described in the supplier’s protocol. The cDNA was placed at -20^o^C until Real-Time PCR analysis.

Real-time PCR was performed with the ViiA^TM^7 real-time PCR system (Applied Biosystems) using Maxima SYBR Green/ROX qPCR Master Mix (cat. no. K0222, Fermentas Inc.). The sequences for porcine 5-LO, LTAH, LTCS and glyceraldehyde-3-phosphate dehydrogenase (GAPDH) primers were reported previously^21^. The validation for the housekeeping gene GAPDH was performed in accordance with the rules described by Dheda et al. (2004)^54^. The starters were synthesized (Laboratory of DNA Sequencing and Oligonucleotide Synthesis, Warsaw, Poland). The reaction mixture of a 25 µl volume contained 12.5 µl SYBR Green/ROX qPCR Master Mix, 1 µM each of forward and reverse primers and 3 µl of diluted RT product. Real-Time PCR conditions included: initial denaturation (10 min, 95^o^C), followed by 40 cycles of denaturation (15 s, 94 °C), annealing (30 s at 59^o^C for *5*-LO, LTCS and LTAH or 61^o^C for GAPDH) and elongation (60 s, 72^o^C). After each PCR reaction, melting curves were generated by stepwise increases in temperature from 60 °C to 95 °C to ensure that a single product was amplified. The gene expression data obtained from Real-Time PCR were normalized against GAPDH using the (Delta Delta C(T)) method^55^, where the average cycling threshold (Ct) was considered.

### Western blot analysis

The total protein concentration in supernatants obtained after the centrifugation of stromal cells was determined^56^. Protein isolates (20 µg) from these cells were diluted in SDS, a gel-loading buffer, heated (4 min, 95 °C) and separated by 10% SDS-polyacrylamide gel electrophoresis. Separated proteins were electroblotted onto 0.45 μm nitrocellulose membranes (cat. no. 1620115, Bio-Rad) in a transfer buffer. Nonspecific binding sites were blocked by incubation (1.5 h, 21^o^C) with 5% fat-free dry milk in Tris-buffered saline with Tween 20 (cat. no. P1379TBS-T, Sigma). The membranes were incubated (18 h, 4^o^C) with primary polyclonal rabbit anti-human antibodies against 5-LO and LTAH (both in dilution 1:500, cat. no. 160402 and 160250, respectively, Cayman Chemical Co.) and against LTCS (in dilution 1:200, cat. no. sc 20108, Santa Cruz Biotechnology). Subsequently, membranes were incubated (1.5 h, 21 °C) with a secondary alkaline phosphatase-conjugated goat anti-rabbit antibody (in dilution 1:10000, cat. no. NB7349, Novus Biologicals). Immune complexes were visualized using a standard alkaline phosphatase visualization procedure (NBT-BCIP, cat. no. 72091, Sigma). Analyses were made in triplicate. To demonstrate the specificity of the rabbit anti-human 5-LO antibody, a specific binding peptide (cat. no. 360402-200, Cayman Chemical Co.) was used as a negative control (Supplementary Fig. 4). The specificity of LTAH and LTCS antibodies was previously reported for porcine uterus^21,24,25^. The membranes were re-probed with polyclonal rabbit anti-GAPDH antibody (in dilution 1:5000; cat. no. G9545, Sigma). Images were acquired and quantified using a CHEMIDOC Touch Imaging System (Image Lab 5.2, Bio-Rad Laboratories, Hercules, CA, USA).

### EIA method

Concentrations of LTB4, LTC4 and cys-LTs in the incubation medium were estimated using EIA kits (cat. no. 520111, 520211 and 10009291, respectively, Cayman Chemical Co.) in accordance with the manufacturer’s instructions. The standard curve for LTB4, LTC4 and cys-LTs ranged from 1.96 to 1000 pg/ml, from 0.98 to 500 pg/ml and from 7.8 to 1000 pg/ml, respectively. The intra- and inter-assay coefficients of variation were 4.5% and 7.2%, respectively, for LTB4, 4.7% and 6.2%, respectively, for LTC4, and 4.4% and 7.5%, respectively, for cys-LTs.

### MMT method

The viability of stromal cells (control /vehicle-treated/, and after exposure to inflammatory mediators) was estimated by the MTT ((3-[4,5-dimethylthiazol-2-yl]-2,5-diphenyltetrazolium bromide) method using an MTT Cell Proliferation Kit (cat. no. 10009365, Cayman Chemical Co.), in accordance with the manufacturer’s instructions.

### Statistical analysis

Only the results from cells that secreted statistically significantly more PGF2α after exposure to NONOate were taken into account. Statistical significance was estimated by one-way analysis of variance (ANOVA) with Bonferroni`s post-hoc analysis (InStat Graph Pad, San Diego, CA, USA). Real-Time PCR data are presented as log2 to increase the readability of the box plots. Statistical significance differences were defined as *P* < 0.05 (*), *P* < 0.01 (**) and *P* < 0.001 (***).

## Electronic supplementary material

Below is the link to the electronic supplementary material.

Supplementary Material 1

## Data Availability

The datasets used and/or analysed during the current study are available from the corresponding author on reasonable request.
