# Supplementary material for: Biosynthetic pathway for leukotrienes is stimulated by lipopolysaccharide and cytokines in pig endometrial stromal cells
Source: Sci Rep. 2025 Jan 22;15:2806. doi: 10.1038/s41598-025-86787-1 (PMC11754892; doi:10.1038/s41598-025-86787-1)
Supplement: Supplementary file 1 — Supplementary Material 1 [file 41598_2025_86787_MOESM1_ESM.pdf]

Biosynthetic pathway for leukotrienes is stimulated by lipopolysaccharide and cytokines in pig endometrial stromal cells

Barbara Jana, *, Aneta Andronowska, Jarosław Całka & Aleksandra Mówińska

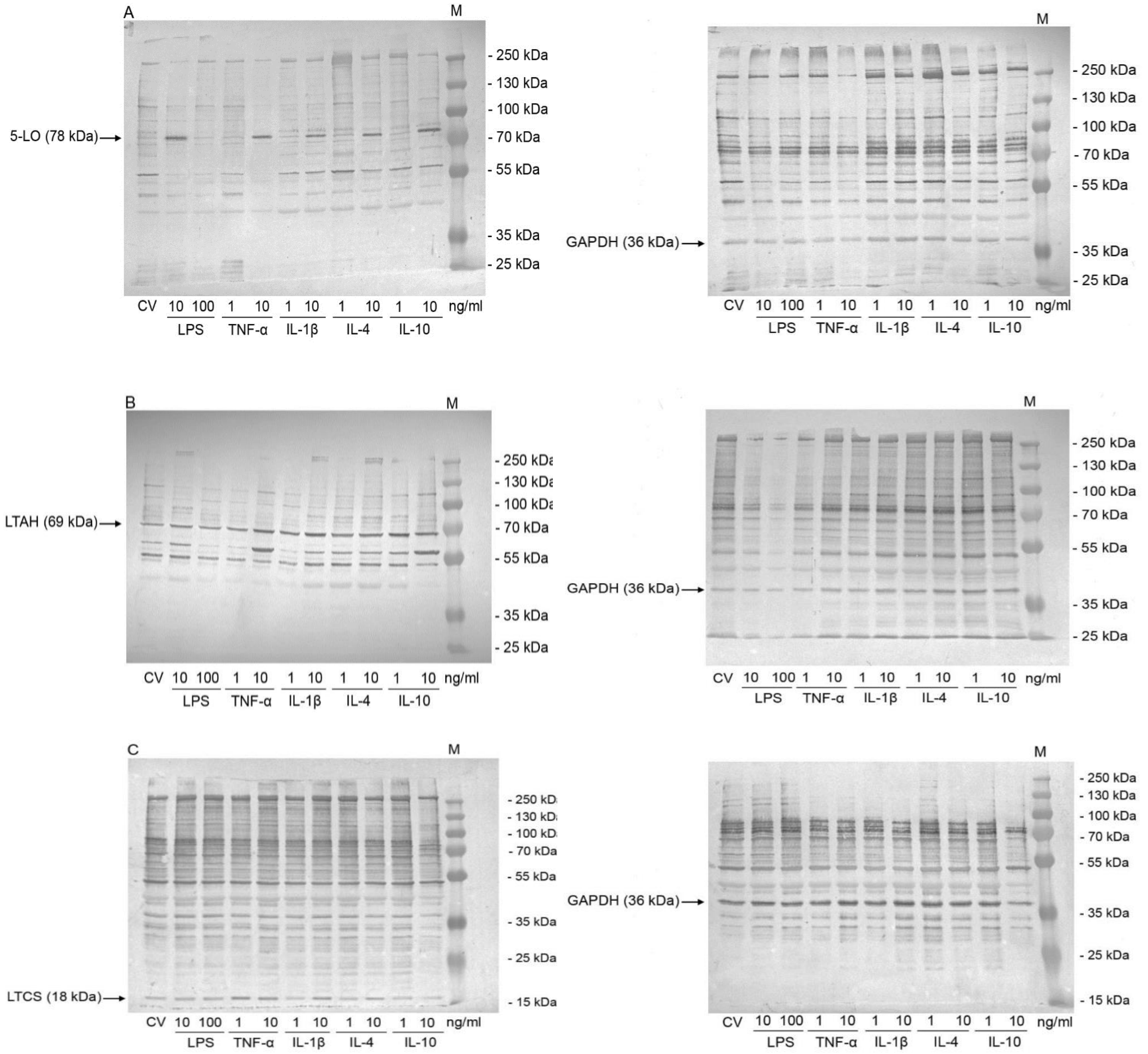

Supplementary Fig. 1. Representative blots demonstrating influence of lipopolysaccharide (LPS), tumor necrosis factor-α (TNF-α), interleukin (IL)-1β, IL-4 and IL-10 on 5-lipoxygenase (5-LO, A), leukotriene A4 hydrolase (LTAH, B) and leukotriene C4 synthase (LTCS, C) protein abundances in the cultured endometrial stromal cells of pigs, determined by Western blotting. Protein values were related to glyceraldehyde-3-phosphate dehydrogenase (GAPDH) protein abundances. For 5-LO antibody bands are visible at 78 kDa, for LTAH antibody at 69 kDa, for LTCS antibody at 18 kDa and for GAPDH antibody at 37 kDa. marker: M - marker; C - control (vehicle-treated cells). All membranes were scanned with the same exposure time.

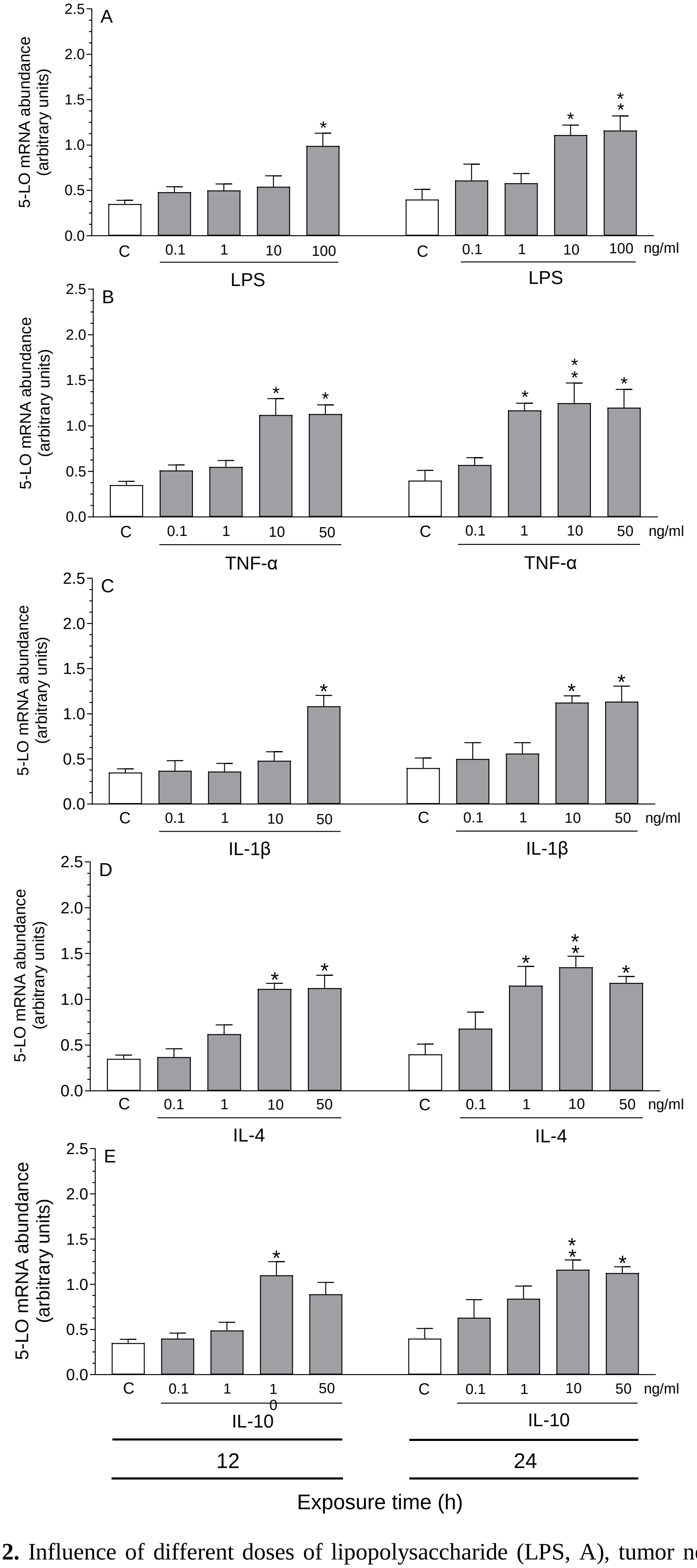

Supplementary Fig. 2. Influence of different doses of lipopolysaccharide (LPS, A), tumor necrosis factor-α (TNF-α, B), interleukin (IL)-1β (C), IL-4 (D) and IL-10 (E) on 5-lipoxygenase (5-LO) mRNA abundances in the cultured endometrial stromal cells of pigs, determined by Real-Time PCR. mRNA values were related to glyceraldehyde-3-phosphate dehydrogenase (GAPDH) mRNA abundances. Treatments were conducted in triplicate for each pig (n=3). Data are presented as the mean ± sem. * (P<0.05), ** (P<0.01) - show statistical differences compared to the control (vehicle-treated cells, C) for 12 h or 24 h exposure period.

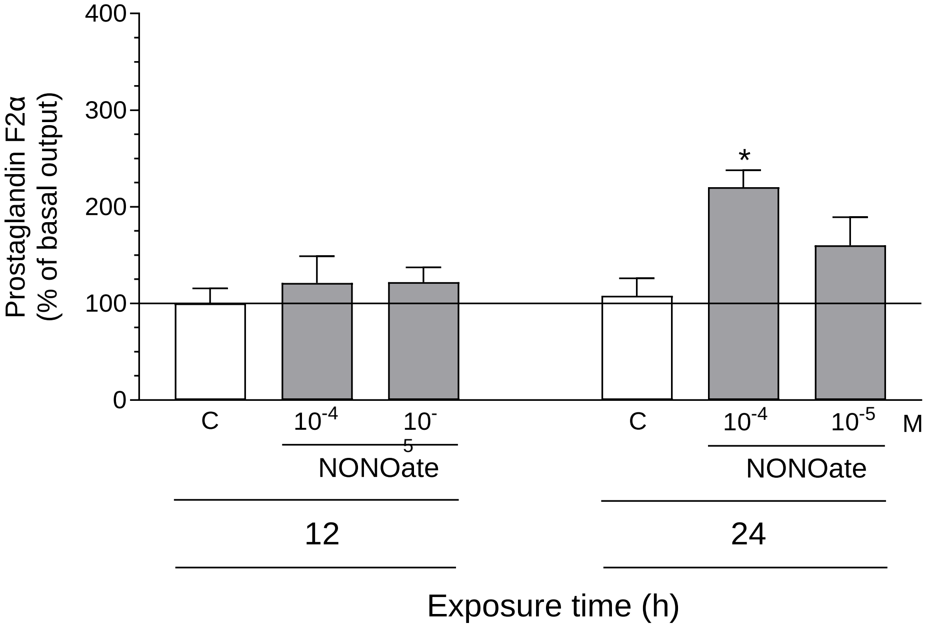

Supplementary Fig. 3. Influence of different doses of nitric oxide donor (NONOate) on prostaglandin F2α (PGF2α) release by the cultured endometrial stromal cells of pigs, determined by ELISA. Treatments were conducted in triplicate for each pig (n=3). Data are presented as the mean ± sem. Values are shown as n-fold change from the control (vehicle-treated cells, C). The PGF2α concentration in the controls were: 14.8 ± 5.3 pg/µg DNA for 12 h exposure, and 19.2±4.8 pg/µg DNA for 24 h exposure. * ($P<0.05$) - shows statistical difference compared to the control for 24 h exposure period.

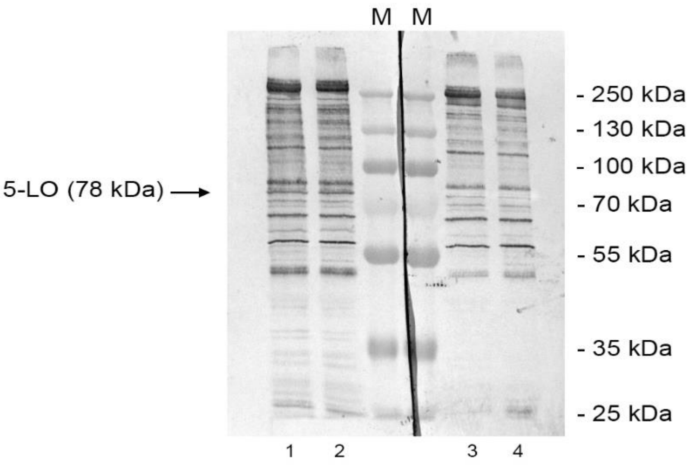

Supplementary Fig. 4. 5-lipoxygenase (5-LO) protein abundance in the endometrial stromal cells of pigs and relevant negative control, determined by Western blotting. Bands for 5-LO antibody at 78 kDa are visible (lines 1 and 2), or much weaker stained after neutralization of primary antibody by specific binding peptide (lines 3 and 4). M - marker. Both parts (left and right) come from the same membrane, samples were electrophoresed and transferred together, and split into two parts before incubation with antibodies. Both parts of the membrane were exposed together, with exactly the same exposure time.
